# Ischemic Stroke in a Seven-Year-Old Female With Down Syndrome and Newly Discovered Moyamoya Syndrome

**DOI:** 10.7759/cureus.45893

**Published:** 2023-09-25

**Authors:** Daniel A Brenner, Elizabeth Flatley, Sushmitha Medappa Maruvanda, Nicholas B Dadario, Daniel J Valdivia, Meenakshi Khosla

**Affiliations:** 1 Neurosurgery, Robert Wood Johnson Medical School, Rutgers University, New Brunswick, USA; 2 Pediatrics, Robert Wood Johnson Medical School, Rutgers University, New Brunswick, USA; 3 Pediatrics, Saint Peter’s University Hospital, New Brunswick, USA

**Keywords:** moyamoya disease, moyamoya syndrome, pediatric moyamoya syndrome, stroke, down syndrome (ds), cerebrovascular, pediatrics, moyamoya

## Abstract

Moyamoya represents a rare, progressive cerebrovascular disease, characterized by a gradual stenosis of the intracranial internal carotid arteries, thereby increasing the risk of stroke. Down syndrome is known to be a predisposing factor for Moyamoya syndrome. This review discusses a distinctive case of a seven-year-old female with Down syndrome who manifested with Moyamoya syndrome, evident from acute stroke-like symptoms.

## Introduction

Moyamoya, a rare yet significant cerebrovascular condition, is defined by the gradual and progressive stenosis of the intracranial internal carotid arteries. This, in turn, induces the formation of compensatory arterial collaterals at the base of the brain [1,2]. Accounting for 6% of childhood strokes, individuals afflicted with moyamoya are considerably susceptible to cerebrovascular accidents [3].

The condition can manifest in two distinct forms: moyamoya disease (MMD) and moyamoya syndrome (MMS). MMD refers to idiopathic instances of the disease, occurring without associated risk factors. In contrast, MMS describes the manifestation of the condition in conjunction with other risk factors, such as neurofibromatosis type 1, sickle cell disease, Down syndrome, or in response to head and neck radiotherapy [2].

Epidemiologically, the incidence of moyamoya shows significant disparities across different ethnicities. The highest prevalence rates have been noted among Asian/Pacific Islander populations (0.509 per 100,000 people), with the second highest incidence observed among Black individuals (0.292 per 100,000). Comparatively, the incidence in White and Hispanic individuals is lower at 0.148 and 0.121 per 100,000 people, respectively [4].

Clinical manifestations of moyamoya are primarily resultant of brain ischemia, with 50-75% of patients initially presenting with either a stroke or a transient ischemic attack [2]. Some patients may also exhibit symptoms attributable to ischemic compensatory responses such as headaches from dilated collateral vessels, or hemorrhagic strokes subsequent to the rupture of fragile collateral vessels [5].

In this case report, we describe the case of a seven-year-old female patient with Down syndrome who presented with acute symptoms indicative of a stroke. Magnetic resonance angiography (MRA) imaging elucidated luminal irregularities in the supraclinoid portions of her internal carotid arteries bilaterally, coupled with collateral vessels in the surrounding areas of the supraclinoid internal carotid arteries and bilateral P1 segments. These findings align with the characteristic presentation of moyamoya. When encountering a patient with Down syndrome who exhibits symptoms of an acute stroke, it is critical to maintain a strong index of suspicion for MMS. This enables timely and accurate diagnosis, thereby facilitating the optimization of patient care.

## Case presentation

The patient is a seven-year-old female with Down syndrome who presented to the emergency department with a one-day history of acute-onset, progressive, right-sided weakness in the setting of a three-day history of viral upper respiratory tract symptoms. The patient developed decreased use of her right arm on the morning of the presentation, which progressed to an inability to walk later in the day.

**Figure 1:**
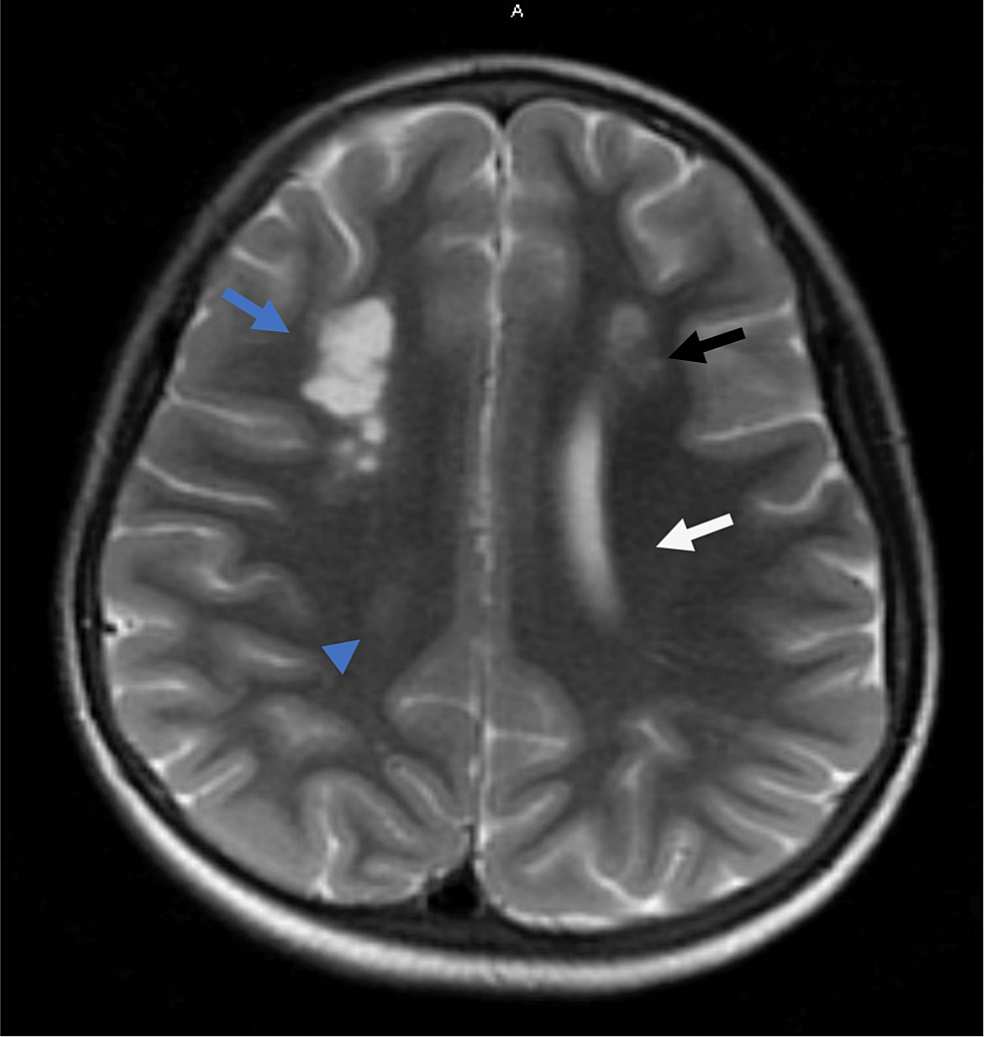
MRI of the brain with areas of restricted diffusion and T2 hyperintensity compatible with acute infarction in the left centrum semiovale (white arrow), left frontal subcortical white matter (black arrow), and, to a lesser extent, right centrum semiovale (blue arrowhead). Areas of chronic infarction can be seen in the right frontal watershed junction (blue arrow).

**Figure 2:**
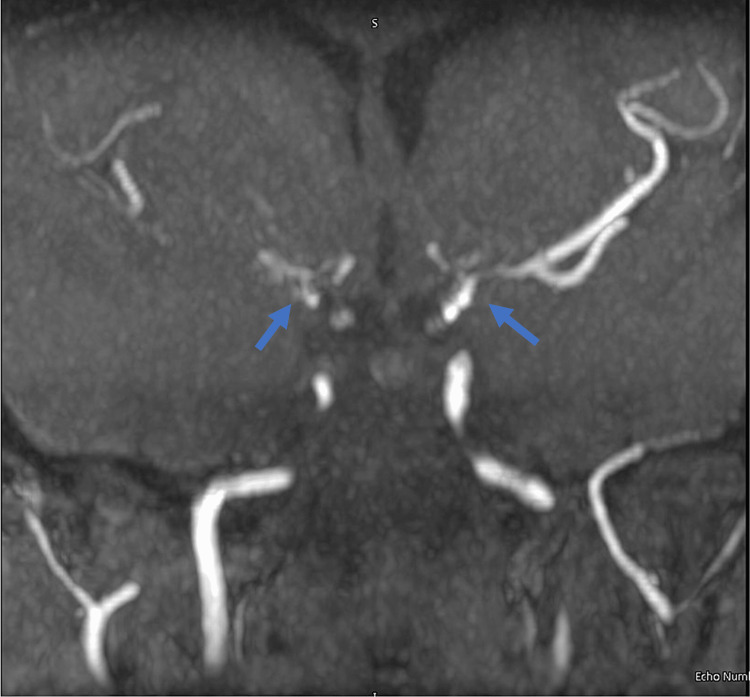
MRA revealing areas of luminal irregularity in the supraclinoid internal carotid arteries bilaterally (blue arrow). Tiny leptomeningeal collateral vessels are regional to the supraclinoid internal carotid arteries and bilateral P1 segments. Findings are compatible with moyamoya.

The initial examination was significant for right hemiparesis with right facial palsy. CT of the head ruled out an acute bleed but showed periventricular hypodensities. Subsequent MRI and MRA revealed an acute left-sided watershed infarct and a chronic right frontal infarct, with vascular stenosis of bilateral internal carotid arteries, consistent with bilateral moyamoya (Figures 1, 2). A lumbar puncture was done to rule out meningitis and revealed clear cerebrospinal fluid (CSF) with a positive meningitis panel for *Streptococcus agalactiae* and enterovirus. She was started on antibiotics for four days but was discontinued after repeat CSF cultures were sterile. The CSF results were deemed contaminated by infectious disease and likely played no role in the patient’s current presentation. Video electroencephalography done for 24 hours ruled out subclinical seizures. MRI of the cervical spine revealed mild cervical straightening, with no evidence of subluxation.

She was managed conservatively, with neuroprotective measures, low-dose aspirin, 1 mg of amlodipine for elevated blood pressure, which peaked at 148/99 mmHg, and physical therapy for rehabilitation. A screening transthoracic echocardiogram showed mild mitral and tricuspid regurgitation, with no vegetation. Over the course of her 18 days in the hospital, the patient’s strength and tone improved significantly, and she was transferred to Children’s Specialized Hospital for continued physical and occupational therapy.

On the two-month follow-up, the patient had regained her strength and was back to her baseline. She was then discharged from the rehabilitation center with no plans for neurosurgical intervention.

## Discussion

The management of moyamoya typically comprises medication and revascularization procedures. There is a paucity of literature concerning the rare presentations, particularly relating to moyamoya and Down syndrome. These conditions have a known connection, yet guidelines for screening and managing this patient demographic are insufficient. Here, we present the case of a seven-year-old female diagnosed with both moyamoya and Down syndrome. Her case underscores the unique diagnostic and management challenges posed by this combination of conditions, especially given the limited literature on the subject.

Despite its rarity, moyamoya is a significant cause of cerebrovascular events in Down syndrome patients, who bear a risk threefold higher than the general population [6,7]. In a comprehensive review by Abdelgadir et al., a total of 54 cases of Down syndrome patients with moyamoya were reported [6]. The demographic most prone to this comorbidity were females aged 12 or under, with symptomatic hemiparesis being the most prevalent clinical manifestation (77%). Speech disorders (22%), seizures (17%), and facial paralysis (11%) were also commonly observed [6]. The case of our patient largely corresponds with this demographic.

Aspirin 81 mg is prescribed for both the acute phase of ischemic infarction and as a preventive measure against further strokes in moyamoya patients. It is also prescribed in the chronic phase as a preventative measure against further strokes. However, surgical revascularization should be considered as an alternative, as it has shown a considerable improvement in prognosis, especially in pediatric cases [8]. Notably, Down syndrome patients have demonstrated excellent responses to this treatment compared to non-Down syndrome patients [9]. The patient discussed here was discharged to a pediatric rehabilitation facility that specialized in chronic neurologically challenged patients and was on daily aspirin as surgery was not deemed necessary.

Considering the evidence put forth by See et al. [9], a diagnosis of moyamoya in Down syndrome patients may often be delayed, potentially explaining the elevated stroke rate in this population. This diagnostic delay could be attributed to several factors. For instance, the developmental delay typical of Down syndrome patients might obstruct their ability to report new neurological deficits. The hemiparesis in our patient, for example, was first noticed by the mother when the patient ceased using her right arm. Furthermore, her speech delay could render it challenging to detect aphasia. There also could have been additional diagnostic imaging conducted such as digital subtraction angiography to help support the diagnosis.

Given the recognized risk of moyamoya in Down syndrome patients, there is a compelling case for the development of robust screening protocols. Protocols already exist for assessing hearing, vision, metabolic syndrome, and thyroid function. Santor et al.’s retrospective study suggested a potential screening method, noting a significant increase in blood pressure up to 18 months before a moyamoya diagnosis in Down syndrome patients [10]. As awareness grows regarding the link between Down syndrome and moyamoya, we hope to see the establishment of comprehensive screening and management protocols, thereby enhancing care for this distinctive patient group.

## Conclusions

Greater awareness of the association between Down syndrome and MMS is essential to improve early detection and diagnosis rates, which is particularly crucial given the potentially serious outcomes such as strokes or seizures associated with moyamoya. It is vital to understand that in patients with Down syndrome, the symptoms of MMS/MMD may be concealed by their underlying condition, thereby possibly delaying diagnosis and treatment. Thus, increased vigilance among healthcare professionals about this association can lead to more timely diagnoses and subsequently improved management and prognosis for this unique patient population.
